# The clinical and cost-effectiveness of corticosteroid injection versus night splints for carpal tunnel syndrome (INSTINCTS trial): an open-label, parallel group, randomised controlled trial

**DOI:** 10.1016/S0140-6736(18)31572-1

**Published:** 2018-10-20

**Authors:** Linda S Chesterton, Milica Blagojevic-Bucknall, Claire Burton, Krysia S Dziedzic, Graham Davenport, Sue M Jowett, Helen L Myers, Raymond Oppong, Trishna Rathod-Mistry, Danielle A van der Windt, Elaine M Hay, Edward Roddy

**Affiliations:** a Arthritis Research UK Primary Care Centre, Research Institute for Primary Care and Health Sciences, Keele University, Keele, UK; b Keele Clinical Trials Unit, Keele University, Keele, UK; c Haywood Academic Rheumatology Centre, Haywood Hospital, Staffordshire and Stoke-on-Trent Partnership NHS Trust, Stoke-on-Trent, UK; d Health Economics Unit, Institute for Applied Health Research, University of Birmingham, Birmingham, UK

## Abstract

**Background:**

To our knowledge, the comparative effectiveness of commonly used conservative treatments for carpal tunnel syndrome has not been evaluated previously in primary care. We aimed to compare the clinical and cost-effectiveness of night splints with a corticosteroid injection with regards to reducing symptoms and improving hand function in patients with mild or moderate carpal tunnel syndrome.

**Methods:**

We did this randomised, open-label, pragmatic trial in adults (≥18 years) with mild or moderate carpal tunnel syndrome recruited from 25 primary and community musculoskeletal clinics and services. Patients with a new episode of idiopathic mild or moderate carpal tunnel syndrome of at least 6 weeks' duration were eligible. We randomly assigned (1:1) patients (permutated blocks of two and four by site) with an online web or third party telephone service to receive either a single injection of 20 mg methylprednisolone acetate (from 40 mg/mL) or a night-resting splint to be worn for 6 weeks. Patients and clinicians could not be masked to the intervention. The primary outcome was the overall score of the Boston Carpal Tunnel Questionnaire (BCTQ) at 6 weeks. We used intention-to-treat analysis, with multiple imputation for missing data, which was concealed to treatment group allocation. The trial is registered with the European Clinical Trials Database, number 2013-001435-48, and ClinicalTrial.gov, number NCT02038452.

**Findings:**

Between April 17, 2014, and Dec 31, 2016, 234 participants were randomly assigned (118 to the night splint group and 116 to the corticosteroid injection group), of whom 212 (91%) completed the BCTQ at 6 weeks. The BCTQ score was significantly better at 6 weeks in the corticosteroid injection group (mean 2·02 [SD 0·81]) than the night splint group (2·29 [0·75]; adjusted mean difference −0·32; 95% CI −0·48 to −0·16; p=0·0001). No adverse events were reported.

**Interpretation:**

A single corticosteroid injection shows superior clinical effectiveness at 6 weeks compared with night-resting splints, making it the treatment of choice for rapid symptom response in mild or moderate carpal tunnel syndrome presenting in primary care.

**Funding:**

Arthritis Research UK.

## Introduction

Carpal tunnel syndrome is the most common compression neuropathy affecting the upper limb, which results from entrapment of the median nerve in the carpal tunnel.^1^ Carpal tunnel syndrome adversely affects daily activities, limits work capacity, and impacts on general health and quality of life.^2^, ^3^ In a primary care population, prevalence has been reported to be 36·08 per 10 000 people, with an annual incidence of 19·12 per 10 000 for men and 35·95 per 10 000 for women.^4^

Classically, carpal tunnel syndrome causes discomfort, paraesthesia, and numbness in the median nerve distribution. Nocturnal symptoms are often clinically significant causing sleep disturbance, and bilateral symptoms occur in more than 50% of patients.^2^ In primary care, diagnosis is based on clinical history and examination findings,^5^ with electrophysiological diagnostic tests requested by the clinician when a clinical diagnosis cannot be determined and when surgical management is considered for severe cases.^6^

No consensus exists for the best primary care management for mild or moderate carpal tunnel syndrome. Mainstay treatments, supported by clinical guidelines,^7^, ^8^ include night-resting splints and local corticosteroid injection. In severe cases or those cases that do not improve with conservative treatment, surgery is considered the treatment of choice.

Systematic review evidence cites two studies, both assessed as low quality, which suggest night splints might be more effective than no active treatment in the short term.^9^ Trials of effectiveness of splint over other non-surgical interventions in the longer term have not been identified. Systematic reviews of corticosteroid injection^10^, ^11^ and a subsequent trial^12^ show strong evidence of effectiveness in the short term (<1 month in the reviews and 10 weeks in the trial) compared with placebo, but evidence for the long-term effects is insufficient.

**Research in context**

**Evidence before this study**

We searched MEDLINE, Embase, CINAHL, and Web of Science for articles published from Jan 1, 1980 to Aug 2, 2011 (updated on Nov 6, 2012 from 2008–12). Before this study, evidence for the comparative clinical or cost-effectiveness of night splints and corticosteroid injection in the medium term or in a primary care population was not robust. At the time of designing the INSTINCTS trial (2009–12), systematic review evidence for the treatment of carpal tunnel syndrome identified only one, small trial, done in a secondary care setting, of head-to-head comparison for two of the mainstay interventions (corticosteroid injections and night-resting splints) used in primary care for this syndrome. A lack of robust evidence of clinical effectiveness beyond the short term for both interventions in any settings was also identified with most existing trials done in secondary care, where patients are likely to have carpal tunnel syndrome that is of longer duration and less responsive to conservative management. No trials had assessed cost-effectiveness of either intervention.

**Added value of this study**

To our knowledge, our study is the largest trial undertaken to date of corticosteroid injection compared with night splints for carpal tunnel syndrome. We have shown clinically significant short-term and sustained improvements for corticosteroid injections compared with night splints, with no safety issues. This is the first study to carry out a full economic assessment and show that both interventions are relatively inexpensive, with corticosteroid injection proving cost-effective over the use of night splints over 6 months.

**Implications of all the available evidence**

In a primary care setting where most treatment for mild or moderate carpal tunnel syndrome is undertaken, the evidence now shows that a corticosteroid injection is the cost-effective treatment of choice for rapid and sustained symptom response compared with night splints. These findings provide evidence to support treatment decision making for policy makers, payers (commissioners), general practitioners, and clinicians in musculoskeletal services, and to offer choice to patients.

The comparative clinical effectiveness of corticosteroid injection and nocturnal wrist splints has only been investigated in two small trials,^13^, ^14^ neither of which were done in a primary care setting. In patients with carpal tunnel syndrome recruited from hospital clinics and confirmed by nerve conduction studies, within-group improvements in symptoms and electrophysiological findings were seen after 11 months of adherent use of nocturnal wrist splints (n=28) but not after a single corticosteroid injection (n=57).^13^ So and colleagues^14^ investigated between-group comparisons 4 weeks after treatment and found that a local corticosteroid injection and nocturnal wrist splints (n=25 participants per group) were equally effective for improving symptom severity, although finger dexterity was improved with local corticosteroid injection.

In the INSTINCTS trial (INjection versus SplinTing in Carpal Tunnel Syndrome), we aimed to investigate the comparative clinical and cost-effectiveness of corticosteroid injection and night splints in primary care.

## Methods

### Study design and participants

We did a pragmatic, two-arm parallel group, open-label, randomised controlled trial within the National Health Service (NHS) in 25 primary and community musculoskeletal clinics and services in England. The trial was approved by the National Research Ethics Service Committee North West—Liverpool (UK; reference 13/NW/0280) and the Medicines and Healthcare products Regulatory Agency (European Clinical Trials Database, number 2013-001435-8). The full protocol has been published before.^15^

We recruited participants attending general practices (via their general practitioner [GP]) and community musculoskeletal clinics (via the treating clinician). Potentially eligible patients were provided with a verbal introduction to the trial and information leaflet by the GP or research clinician. Patients were eligible for inclusion if they were aged 18 years or older and presented with a new episode of primary idiopathic mild or moderate carpal tunnel syndrome, which had been present for longer than 6 weeks. A GP or trained clinician (physiotherapist or occupational therapist) made the clinical diagnosis, standardised on the basis of presenting symptoms, clinical history, and physical tests using criteria developed as part of a consensus survey of GPs from the UK Primary Care Rheumatology Society.^16^ Mild carpal tunnel syndrome was defined as intermittent paraesthesia in the distribution of the median nerve and moderate as constant paraesthesia, and reversible numbness or pain of idiopathic nature.^17^ Participants with bilateral carpal tunnel syndrome designated their study hand on the basis of the most severe symptoms. We excluded participants if they had severe carpal tunnel syndrome exhibiting constant wrist and hand (specifically palm, index, or middle finger, or thumb) pain, numbness or sensory loss in the wrist and hand (specifically palm, index, or middle finger, or thumb), or thenar muscle atrophy; had received a corticosteroid injection or night splint for carpal tunnel syndrome within the preceding 6 months; had previous surgery in the affected wrist, trauma to the affected hand requiring surgery, or immobilisation in the previous 12 months; had current or previous infection of the affected wrist, local or systemic sepsis or infection, or intercurrent illness; were pregnant or lactating; were in receipt of anticoagulants; had a history of hypersensitivity to methylprednisolone acetate or any of its excipients; were allergic to any of the splint materials; had a history of drug or alcohol abuse; were undergoing ongoing litigation; or were unable to complete self-report questionnaires written in English. Written, informed consent was obtained from eligible participants who were interested in taking part in the trial.^15^

### Randomisation and masking

Participants were randomly assigned (1:1) to either treatment group with permutated blocks of sizes two and four, prestratified by research site. Randomisation was completed by the Keele University (Keele, UK) Clinical Trial Unit's (CTU) online web or telephone randomisation service. The allocation sequence was not available to research team members. We could not mask treating clinicians or patients to treatment allocation, but we concealed the treatment group allocation during the analyses. A letter was sent to the GPs of all participants informing them of their patient's participation in the trial and their treatment allocation.

### Procedures

Participants recruited via GP consultation received the trial intervention over two appointments. Those participants recruited after being sent trial information by post received the trial intervention at one appointment.^15^

Participants randomly assigned to receive corticosteroid injection received one injection of 20 mg methyl-prednisolone acetate (as 20 mg of Depo-Medrone from 40 mg/mL; Pfizer Manufacturing Belgium NV; Puurs, Belgium) via a disposable needle (23G or 25G) and syringe which was inserted at the wrist between the proximal and distal wrist crease to infiltrate the carpal tunnel. We did not allow injections into the palm of the hand. Patients were treated by the diagnosing clinician who used a sterile no-touch technique without local anaesthetic. Participants were advised to wait for 30 min following injection and to rest the injected arm for 48 h. They were given two Arthritis Research UK patient leaflets for carpal tunnel syndrome and local corticosteroid injections.^18^, ^19^

Participants randomly assigned to a resting night splint received a Beta Wrist Brace (with CE marking; Promedics Orthopaedic; Port Glasgow, UK), which immobilised the wrist in a neutral or slightly extended position (20° from neutral) intended to reduce pressure within the carpal tunnel, to wear at night for 6 weeks. The splint was fitted according to the size of the participant's hand and arm with standard splints of differing sizes. The treating clinician showed the participants how to fit and remove the wrist splint and gave them two Arthritis Research UK patient leaflets: carpal tunnel syndrome and splints for arthritis of the hand and wrist.^20^ The clinician instructed the participants to do gentle range-of-motion exercises when removing the splint to prevent stiffness and reinforced adherence by verbal instruction.

No other types of therapy in either group were advised during the first 6 weeks, except for simple analgesia either prescribed or bought over the counter (paracetamol and non-steroidal anti-inflammatory drugs). To preserve the pragmatic nature of the trial, participants with bilateral carpal tunnel syndrome were permitted treatment for the non-study hand according to normal clinical protocols in use at the research site.

Baseline data were collected from a self-completed questionnaire immediately before randomisation. All outcome measures were also collected at 6 weeks, as the timepoint when further treatment is most likely to be considered in primary care, and 6 months by postal self-complete questionnaire, with the exception of self-reported adverse events, which were collected in the 6-week questionnaire only. Non-responders to follow-up questionnaires were sent a reminder postcard after 2 weeks. Those individuals who did not respond after the reminder postcard were sent a repeat questionnaire after a further 2 weeks. Non-responders to the repeat questionnaire reminders were telephoned by the research nurse, who was masked to the treatment group, to collect the primary outcome measure. Participants who had not been successfully contacted by telephone after five attempts were sent a postal minimum data questionnaire.

### Outcomes

The primary outcome was the overall score for symptom severity and limitations in hand function on the Boston Carpal Tunnel Questionnaire (BCTQ) at 6 weeks (appendix).^21^ The BCTQ is a disease-specific questionnaire referring to a typical 24-h period in the last 2 weeks of completing the questionnaire, which was done at baseline and all follow-up points. It consists of two subscales: symptom severity scale (11 items) and function status scale (eight items). Secondary outcome measures at 6 weeks, 6 months, 12 months, and 24 months included BCTQ symptom severity and function status subscales,^21^ hand–wrist symptom intensity (0–10 numerical rating scale), referral for surgery, surgery, and self-reported adherence. Secondary outcome measures at 6 weeks and 6 months only included interrupted sleep.^22^ Secondary measures at 6 months, 12 months, and 24 months only were over-the-counter and prescribed analgesia, perceived benefit and satisfaction with treatment,^23^ impact of carpal tunnel syndrome on work and activities, general health (EuroQoL EQ-5D-5L),^24^ health-care use and patient incurred costs, and use of co-interventions. Furthermore, performance at work and days off work were also measured at all follow-up timepoints and were used in sensitivity analyses (appendix). Long-term follow-up of 12 months and 24 months will be published elsewhere once collection is complete in early 2019.

Incident adverse events from either intervention were reported and assessed with clinical case report forms, participant self-report in follow-up questionnaires or directly to the CTU, or to their GP.

### Statistical analysis

We aimed to detect a 15% greater improvement on the BCTQ from an expected baseline value of about 2·9 points (scale 1–5 [SD 1·0])^12^, ^25^, ^26^, ^27^ in the corticosteroid injection group compared with night splinting (ie, a 0·9-point (30%) reduction in the injection group versus a 0·45-point (15%) reduction in the night splint group, with a pooled SD of 1·0 and standardised mean difference of 0·45). Given 90% power, 5% two-tailed significance, and assuming 15% loss to follow-up, 240 patients (120 in each treatment group) were required.

We used descriptive statistics (mean and SD or frequency counts and percentages, as appropriate) to summarise baseline characteristics of participants by treatment group and assess similarity of randomised participants with eligible non-participants. The primary analysis was by intention to treat and was done independently by two statisticians, who remained masked to treatment allocation up until the per-protocol analysis. Multiple imputation with chain equations^28^ were used to impute missing data arising from questionnaire non-response or non-completion of all items in the questionnaire. Data were assumed to be missing at random. Multiple imputation was applied to all participants randomly assigned to treatment, and the number of imputations was set at 35. Results based on multiply imputed data were compared with those based on complete-case analysis as a sensitivity analysis.

The primary between-group evaluation used multiple linear regression to obtain the mean between-group difference with 95% CI in the BCTQ overall score at 6 weeks, adjusting for age at randomisation, sex, duration of symptoms, and baseline BCTQ-score. As a sensitivity analysis, if an imbalance existed in patient characteristics between the treatment groups, the affected characteristics would be further adjusted for.

By use of linear and logistic regression models for continuous and dichotomous outcomes respectively, secondary analyses included between-group comparisons of the BCTQ overall score at 6 months, the BCTQ symptom severity and function subscales, and other outcome measures recorded at 6 weeks or 6 months, or both.

We did a per-protocol sensitivity analysis based on self-reported adherence to night splinting for at least 4–6 nights per week and use of a single corticosteroid injection. No interim analyses were completed.

Potential effect modification was investigated for participants' expectations of the probable response to corticosteroid injection or night-splinting treatments as recorded at baseline and presence of bilateral carpal tunnel syndrome, and analysed through adding moderator × treatment interactions to the models estimating the primary outcome of overall BCTS score, to provide exploratory findings regarding subgroup effects at 6 weeks and 6 months.

The primary economic analysis was a cost-utility analysis, done from an NHS perspective, to determine the cost-effectiveness of night splints versus corticosteroid injection. Health-care resource use data were obtained from self-report questionnaires at 6 months and these were valued with unit cost data obtained from the British National Formulary,^29^ Unit Costs of Health and Social Care,^30^ and NHS reference costs.^31^ We also estimated the cost of delivering both interventions (night splints and corticosteroid injections). All costs were valued at 2016–17 prices. Outcomes were measured in quality-adjusted life-years (QALYs). We calculated QALYs over 6 months for each study participant using EQ-5D 5L scores and the area under the curve approach. Imbalances in baseline utility (EQ-5D) scores between the study arms were controlled for with a multiple linear regression approach. Missing cost and EQ-5D-5L scores were imputed with a multiple imputation approach. An imputation model was fitted and included 25 imputed datasets.

An incremental analysis was undertaken, with differences in costs and QALYs expressed as an incremental cost-effectiveness ratio (ICER) of cost per additional QALY gained. Bootstrapping was used to quantify uncertainty, and 5000 paired estimates of mean differential costs and QALYs were estimated and presented on a cost-effectiveness plane. A cost-effectiveness acceptability curve was constructed to show the probability of injection being cost-effective across a range of possible values of willingness to pay for an additional QALY.

Sensitivity analysis had four main foci. First, broader societal costs were calculated with the human capital approach to determine productivity losses due to time off work over the 6-month period. Costs of absenteeism from paid work were estimated by multiplying the reported number of days off work by the average daily wage, stratified by hourly mean income according to sex, full-time or part-time work status, and standard occupational classification (2010).^32^ Second, sensitivity analysis focused on estimating the cost-effectiveness of the interventions from a health-care perspective (including private health-care costs). Third, cost-utility analysis was done with individual-level utility scores obtained with the EQ-5D-5L questionnaire mapped back to the EQ-5D 3L valuation set, as recommended by National Institute for Health and Care Excellence.^33^ Finally, cost-effectiveness analysis was done with the BCTQ. Results were deemed statistically significant if p values were less than 0·05. We did all analyses using Stata 13.

An external trial steering and data monitoring committee were appointed. The trial was prospectively registered with ClinicalTrials.gov on Jan 16, 2014, (NCT02038452) and registered with Current Controlled Trials on May 1, 2014 (ISRCTN09392969).

### Role of the funding source

The funder had no role in study design, data collection, data analysis, data interpretation, or writing of the report. The lead author (LSC) and data custodian (TR-M) had full access to all data in the trial and LSC had final responsibility for the decision to submit for publication.

## Results

**Figure 1:**
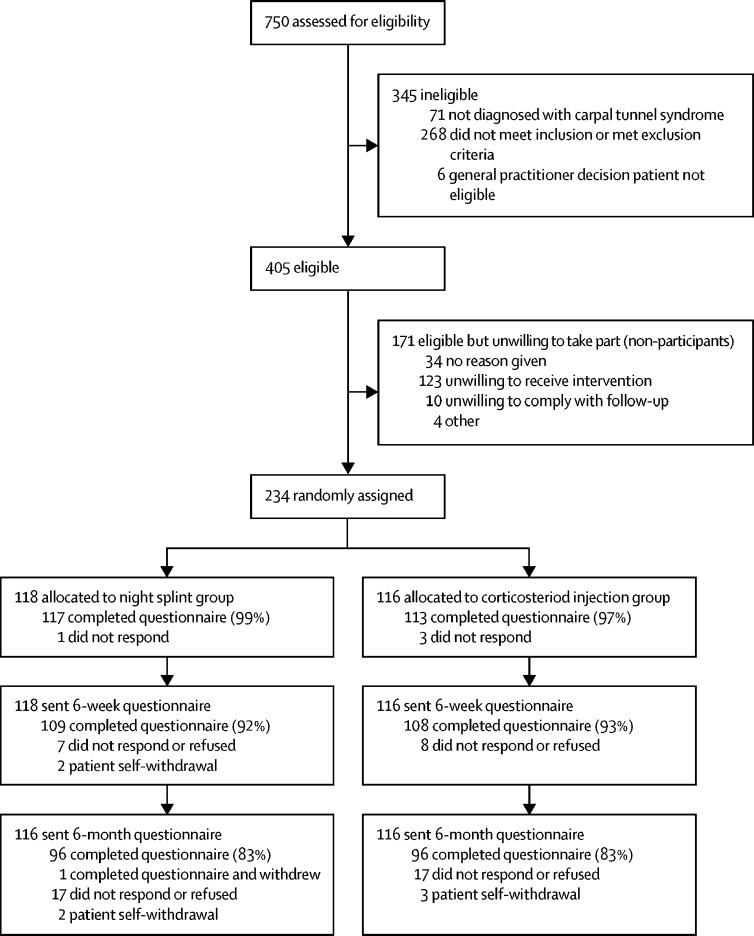
Consort flow diagram

Between April 17, 2014, and Dec 31, 2016, 750 patients were seen at their general practice or community musculoskeletal clinic and assessed for eligibility, of whom 405 (54%) fulfilled eligibility criteria (figure 1). The most frequent reasons for ineligibility were that patients had received corticosteroid injection or night splints in the preceding 6 months (n=117, 26%), had severe carpal tunnel syndrome symptoms (n=54, 12%), and had intercurrent illnesses (n=53, 12%). Of the eligible patients, 234 gave informed consent and were randomly assigned to treatment (58% of 405 eligible patients and 31% of 750 clinical attendees); 118 to the night splint group and 116 to the corticosteroid injection group. The number of participants randomly assigned at each centre ranged from 0 to 36. Demographic characteristics were similar between participants (n=234) and eligible non-participants (n=171): mean age 52·4 years (SD 15·9) versus 53·4 years (14·7) and n=80 (34%) versus n=50 (29%) were men, respectively. Clinicians reported 22 protocol deviations (15 treatment, one eligibility, three data capture, two recruitment, and one pharmacovigilance) and participants reported 11 self-report adherence deviations.

Most baseline characteristics were similar between treatment groups, with minor imbalances; however, larger imbalances were observed for currently being in a paid job, presence of other conditions affecting the neck, shoulders or elbow, and how the hand or wrist problems started (table 1). 217 participants returned the 6-week questionnaire, exceeding the required sample size for the primary analysis of n=200 (figure 1). 193 participants (one withdrew) returned the 6-month questionnaire (figure 1). Participants lost to follow-up at 6 weeks tended to be younger (mean age 53·3 years [SD 15·8)] for responders and 40·6 years [13·4] for non-responders), more often men (n=72 [33%] *vs* n=8 [47%]), and in employment (n=121 [56%] *vs* n=11 [65%]; appendix).

**Table 1:** Baseline characteristics

		**Corticosteroid injection group (n=116)**	**Night splint group (n=118)**
**Demographics**			
Age			
	Mean (SD)	52·6 (17·0)	52·2 (14·9)
	Median (IQR)	53·50 (39·25–65·00)	50·00 (40·75–64·25)
Sex			
	Male	43 (37%)	37 (31%)
	Female	73 (63%)	81 (69%)
**Previous history of carpal tunnel syndrome**			
First time diagnosed with carpal tunnel syndrome			
	Yes	97 (84%)	102 (86%)
	No	16 (14%)	15 (13%)
	Missing	3 (3%)	1 (1%)
Number of times previously had carpal tunnel syndrome			
	Not applicable	97 (84%)	102 (86%)
	1	11 (9%)	8 (7%)
	2	1 (1%)	1 (1%)
	3	0	0
	>3	3 (3%)	4 (3%)
	Missing	4 (3%)	3 (3%)
Treatments for previous carpal tunnel syndrome for either hand			
	Not applicable	97 (84%)	102 (86%)
	None	5 (4%)	8 (7%)
	Steroid injection	3 (3%)	4 (3%)
	Wrist splints	7 (6%)	4 (3%)
	Carpal tunnel decompression (surgery)	3 (3%)	2 (2%)
	Ultrasound	1 (1%)	1 (1%)
	Exercises	0	0
	Vitamin supplements	0	1 (1%)
	Changes in the work place	0	0
	Physiotherapy	1 (1%)	0
**Current carpal tunnel syndrome**			
Which problematic hand or wrist?			
	Right	36 (31%)	37 (31%)
	Left	19 (16%)	20 (17%)
	Both	57 (49%)	59 (50%)
	Missing	4 (3%)	2 (2%)
If both hands problematic, which hand is worse?			
	Not applicable	55 (47%)	57 (48%)
	Right	25 (22%)	24 (20%)
	Left	13 (11%)	19 (16%)
	No difference	7 (6%)	3 (3%)
	Missing	16 (14%)	15 (13%)
Duration of hand or wrist problems			
	<3 months	19 (16%)	17 (14%)
	3–6 months	37 (32%)	33 (28%)
	6 months to 1 year	22 (19%)	27 (23%)
	>1 year	34 (29%)	39 (33%)
	Missing	4 (3%)	2 (2%)
How did hand or wrist problems start?			
	Suddenly: symptoms developed quickly within a few days	33 (28%)	17 (14%)
	Gradually: symptoms developed more slowly over weeks to months	79 (68%)	99 (84%)
	Missing	4 (3%)	2 (2%)
Particular position causes hand or wrist problems			
	Yes	50 (43%)	62 (53%)
	No	62 (53%)	54 (46%)
	Missing	4 (3%)	2 (2%)
Currently taking pain relief for hand or wrist problems			
	Yes	36 (31%)	34 (29%)
	No	77 (66%)	83 (70%)
	Missing	3 (3%)	1 (1%)
**Employment**			
In a current paid job			
	Yes	58 (50%)	74 (63%)
	No	55 (47%)	42 (36%)
	Missing	3 (3%)	2 (2%)
If not in current paid job, describe current situation			
	Not applicable	58 (50%)	74 (63%)
	Retired	38 (33%)	29 (25%)
	Student	3 (3%)	1 (1%)
	Looking after children or home	12 (10%)	9 (8%)
	Unemployed	3 (3%)	7 (6%)
	Voluntary worker	2 (2%)	0
	Missing	3 (3%)	2 (2%)
**Treatment expectations**			
Which treatment would you prefer?			
	Strongly prefer wrist injection	13 (11%)	13 (11%)
	Somewhat prefer wrist injection	11 (9%)	21 (18%)
	No preference	65 (56%)	60 (51%)
	Somewhat prefer night splints	12 (10%)	8 (7%)
	Strongly prefer night splints	6 (5%)	10 (8%)
	Missing	9 (8%)	6 (5%)
If you received a wrist injection, would you expect your symptoms to improve?			
	Yes	69 (59%)	70 (59%)
	No	0	2 (2%)
	Not sure	39 (34%)	41 (35%)
	Missing	8 (7%)	5 (4%)
If you received a night splint, would you expect your symptoms to improve?			
	Yes	46 (40%)	40 (34%)
	No	3 (3%)	4 (3%)
	Not sure	58 (50%)	69 (58%)
	Missing	9 (8%)	5 (4%)
**Quality of life**			
Have you been bothered by feeling down, depressed, or hopeless?			
	Yes	47 (41%)	39 (33%)
	No	66 (57%)	78 (66%)
	Missing	3 (3%)	1 (1%)
Have you been bothered by little interest or pleasure in doing things?			
	Yes	39 (34%)	33 (28%)
	No	74 (64%)	83 (70%)
	Missing	3 (3%)	2 (2%)
**General health**			
Diagnosed with hypothyroidism			
	Yes	5 (4%)	9 (8%)
	No (negative or never tested)	108 (93%)	107 (91%)
	Missing	3 (3%)	2 (2%)
Diagnosed with diabetes			
	Yes	13 (11%)	8 (7%)
	No	99 (85%)	109 (92%)
	Missing	4 (3%)	1 (1%)
Any other conditions affecting neck, shoulders, or elbows			
	Yes	45 (39%)	28 (24%)
	No	68 (59%)	88 (75%)
	Missing	3 (3%)	2 (2%)
Had pain anywhere else apart from your hand or wrist			
	Yes	74 (64%)	72 (61%)
	No	39 (34%)	45 (38%)
	Missing	3 (3%)	1 (1%)
Last time you were free of pain anywhere			
	<3 months ago	22 (19%)	20 (17%)
	3–6 months ago	13 (11%)	19 (16%)
	6 months to 1 year ago	9 (8%)	23 (19%)
	1–3 years ago	38 (33%)	23 (19%)
	>3 years ago	31 (27%)	32 (27%)
	Missing	3 (3%)	1 (1%)
On average, how often do you drink alcohol?			
	Daily or most days	11 (9%)	11 (9%)
	Once or twice a week	38 (33%)	45 (38%)
	Once or twice a month	25 (22%)	22 (19%)
	Once or twice a year	16 (14%)	18 (15%)
	Never	23 (20%)	21 (18%)
	Missing	3 (3%)	1 (1%)
What is your current smoking status?			
	Never smoked	55 (47%)	55 (47%)
	Previously smoked	39 (34%)	48 (41%)
	Current smoker	19 (16%)	13 (11%)
	Missing	3 (3%)	2 (2%)
	Mean body-mass index (SD)	30·2 (7·6)	30·5 (7·5)

Data are n (%), unless otherwise stated. Data might not total 100% because of rounding.

At 6 weeks, a significantly greater improvement in overall BCTQ score was seen for the corticosteroid injection group (mean score 2·02 [SD 0·81]) than for the night splint group (2·29 [0·75]; adjusted mean difference −0·32; 95% CI −0·48 to −0·16; p=0·0001; table 2). Adjusted effect estimates for BCTQ symptom severity subscale, BCTQ functional status subscale, hand–wrist pain intensity, and insomnia due to hand or wrist problems were better or were less frequent in the injection group than the night splint group at 6 weeks (table 2).

**Table 2:** Comparative treatment effectiveness at 6 weeks and 6 months

		**Corticosteroid injection group (n=116)**	**Night splint group (n=118)**	Regression analysis*	
				Adjusted mean difference (95% CI) or OR (95% CI)	p value
Overall BCTQ symptom severity and functional limitations					
	Baseline	2·69 (0·70)	2·65 (0·62)	..	..
	6 weeks†	2·02 (0·81)	2·29 (0·75)	−0·32 (−0·48 to −0·16)‡	0·0001
	6 months	2·15 (0·79)	2·06 (0·73)	0·06 (−0·11 to 0·23)	0·499
BCTQ symptom severity scale					
	Baseline	2·96 (0·66)	2·91 (0·61)	..	..
	6 weeks	2·12 (0·84)	2·43 (0·76)	−0·35 (−0·53 to −0·17)	0·0001
	6 months	2·33 (0·86)	2·18 (0·75)	0·13 (−0·07 to 0·33)	0·209
BCTQ functional limitations					
	Baseline	2·32 (0·92)	2·28 (0·84)	..	..
	6 weeks	1·88 (0·88)	2·09 (0·86)	−0·26 (−0·43 to −0·09)	0·0031
	6 months	1·91 (0·84)	1·89 (0·84)	−0·005 (−0·175 to 0·166)	0·957
Hand–wrist pain intensity					
	Baseline	6·33 (2·05)	6·12 (2·21)	..	..
	6 weeks	3·42 (2·77)	4·28 (2·73)	−0·97 (−1·64 to −0·30)	0·0049
	6 months	4·32 (3·26)	3·46 (3·01)	0·79 (−0·02 to 1·59)	0·055
Insomnia due to hand or wrist problems					
	Baseline	70 (60·6%)	60 (50·6%)	..	..
	6 weeks	33 (28·2%)	45 (38·3%)	OR 0·44 (0·22–0·87)	0·018
	6 months	37 (31·9%)	32 (27·2%)	OR 1·12 (0·55–2·20)	0·755
Referral to surgery					
	6 weeks	4 (3·2%)	5 (4·6%)	..§	..§
	6 months	22 (18·6%)	14 (11·9%)	OR 1·66 (0·73–3·77)	0·227
Surgery					
	6 weeks	2 (1·3%)	2 (1·8%)	..§	..§
	6 months	17 (14·3%)	13 (11·1%)	OR 1·28 (0·41–3·98)	0·664
Herbal remedies and vitamin use at 6 months		7 (6·4%)	7 (6·0%)	OR 1·43 (0·28–7·34)	0·664
Over-the-counter pain medication at 6 months¶		34 (29·5%)	30 (25·3%)	OR 1·42 (0·63–3·18)	0·399
Prescribed pain medication at 6 months‖		20 (17·5)	12 (10·6%)	OR 1·99 (0·70–5·66)	0·197

Score range 1–5 (higher score indicates more severe symptoms and functional impairment). Data are mean (SD) or n (%). Analyses are based on multiply imputed data. BCTQ=Boston Carpal Tunnel Questionnaire.

* Regression analysis adjusted for baseline score (if available), sex, age, and duration of symptoms.

† 11% difference in overall BCTQ scores change between baseline and 6 weeks.

‡ Corresponding pooled SD 0·786.

§ Unable to analyse because of too few patients.

¶ Included paracetamol, ibuprofen, and paracetamol and codeine.

‖ Included paracetamol, ibuprofen, naproxen, diclofenac, codeine, tramadol, dihydrocodeine, paracetamol and codeine, paracetamol and tramadol, paracetamol and dextropropoxyphene, and paracetamol and dihydrocodeine.

**Figure 2:**
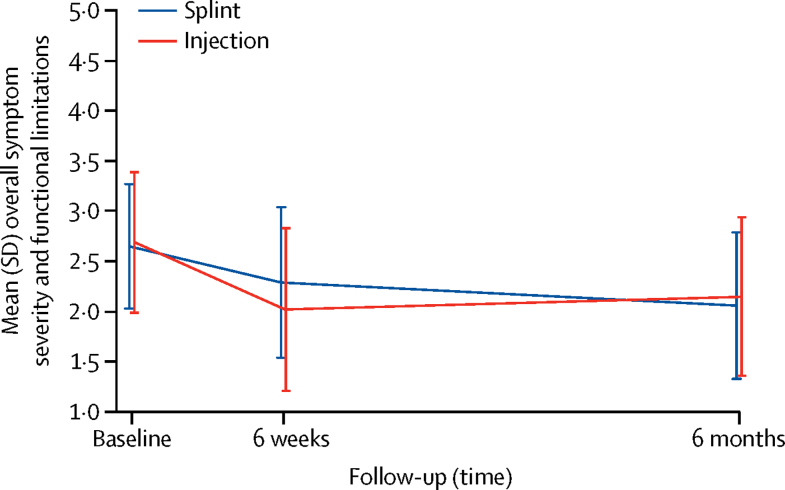
Symptom severity and functional limitations

At 6 months, further improvement in overall BCTQ score was seen in the night splint group, while the corticosteroid injection group on average sustained improvements observed at 6 weeks (figure 2). However, outcomes did not differ between treatment groups (table 2).

No serious or unexpected adverse events were reported. In terms of expected adverse reactions, within the corticosteroid injection group four (3%) participants reported thinning, lightening, or darkening of the skin at the injection site, 17 (15%) had hot flushes, and 53 (46%) had a more painful hand or wrist after the injection, of whom 18 (34%) reported the pain lasting more than 3 days before it started to ease. In the night splint group, seven (6%) were not able to wear the splints as instructed because the splints were uncomfortable. Further adjustment of the analysis of primary outcome for the imbalanced baseline characteristics resulted in negligible changes of the results (adjusted mean difference −0·33, 95% CI −0·51 to −0·16 for the injection group *vs* −0·32, −0·48 to −0·16 for the night splint).

The results of the sensitivity analysis comparing effect estimates based on multiple imputation with complete-case analysis were similar for all outcomes at 6 weeks and 6 months (adjusted mean difference for overall BCTQ score at 6 weeks −0·32, 95% CI −0·48 to −0·16 based on multiple imputation for 234 participants *vs* −0·36, −0·54 to −0·19 based on complete-case analysis of 197 participants; adjusted mean difference for overall BCTQ score at 6 months 0·06, −0·11 to 0·23 based on multiple imputation for 234 participants *vs* 0·05, −0·13 to 0·24 based on complete-case analysis of 175 participants).

For the per-protocol sensitivity analysis, patients whose treatment deviated from protocol were excluded (n=31). In the corticosteroid injection group, three participants were excluded from the analysis as they either received an incorrect injection (n=2) or additionally to the injection wore a night splint (n=1; therefore switched treatment group). In the night splint group, 28 participants were removed from the analysis as they received a corticosteroid injection in addition to the night splint (n=2; therefore switched treatment group), wore the splint on the wrong hand (n=3), did not wear the splint for at least 4–6 nights per week (n=4), or did not provide adherence data (n=19). Therefore, 89 patients in the night splint group and 114 patients in the injection group were included in the per-protocol sensitivity analysis. The results were similar to the intention-to-treat analysis with adjusted mean differences for overall BCTQ scores of −0·36 (95% CI −0·55 to −0·18) versus −0·32 (–0·48 to −0·16; table 2) at 6 weeks and 0·05 (–0·15 to −0·25) versus 0·06 (–0·11 to 0·23; table 2) at 6 months. Between 6 weeks and 6 months after randomisation, in the injection group nine participants were using night splints and in the night splint group 12 participants had received an injection.

Exploratory, a-priori defined subgroup analysis in participants with unilateral and bilateral carpal tunnel syndrome showed no statistically significant or important effect modification on the overall BCTQ score (–0·14, 95% CI −0·47 to 0·19). The adjusted mean difference for overall BCTQ score for injection versus splint was −0·25 (95% CI −0·47 to −0·02) for those with unilateral symptoms and −0·39 (–0·62 to −0·15) for those with bilateral symptoms at 6 weeks. Similar results were obtained at 6 months for the effect modification (–0·15, 95% CI −0·48 to 0·19) and the adjusted mean difference was 0·13 (95% CI −0·10 to 0·37) for those with unilateral symptoms and −0·01 (–0·25 to 0·23) for those with bilateral symptoms.

The exploratory, a-priori defined subgroup analysis investigating effect modification by participants' expectations regarding treatment response showed larger improvements in BCTQ scores were seen in those allocated to the intervention of their preference (n=42, adjusted mean difference −0·52, 95% CI −0·93 to −0·12) compared with those who were not allocated to the intervention of their preference (n=52, −0·12, −0·50 to 0·26). Those individuals who preferred to have an injection showed larger improvements (n=58, −0·60, −0·97 to −0·23) than those who preferred a night splint (n=52, −0·22, −0·60 to 0·16) or had no preference for either treatment (n=128, −0·24, −0·44 to −0·05). For both subgroup analyses, effect modification by treatment expectations was not statistically significant.

**Figure 3:**
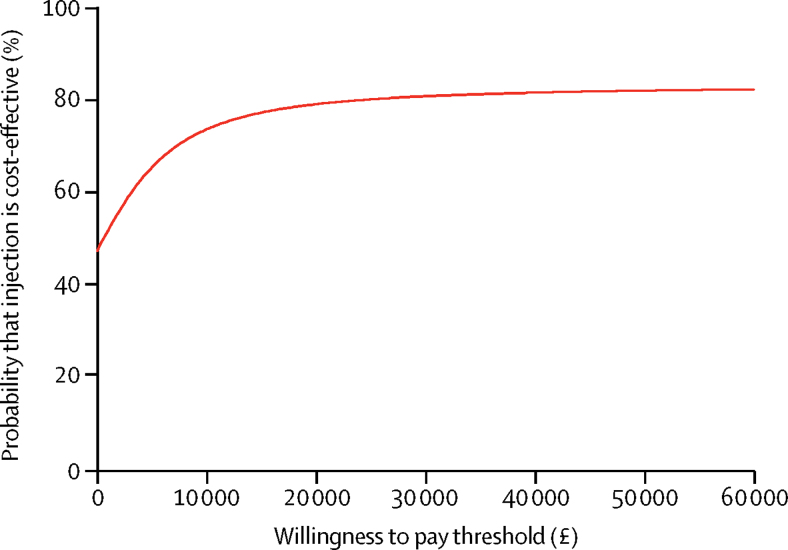
Cost-effectiveness acceptability curve

With regards to the resource use, costs, and outcomes (EQ-5D and QALYs) over 6 months, from an NHS perspective, corticosteroid injection was more costly and more effective (cost difference £33·54, 95% CI −94·57 to 145·59, QALY difference 0·008, 95% CI −0·01 to 0·02) than night splinting (figure 3; appendix). The resulting ICER was £4193 per QALY gained (table 3) with a 76% probability of corticosteroid injection being cost-effective at a willingness-to-pay threshold of £20 000 per QALY gained (figure 3).

**Table 3:** Cost-utility analysis for injection versus splinting

	**Corticosteroid injection group (n=116)**	**Night splint group (n=118)**	**Difference (95% CI)**	**ICER**
Mean costs (SD)	£346·78 (467·97)	£313·24 (480·84)	£33·54 (−94·57 to 145·59)	£4193 per QALY gained
Mean QALYs* (SD)	0·404	0·396	0·008 (−0·01 to 0·02)	..

ICER=incremental cost-effectiveness ratio. QALY=quality-adjusted life-year.

* Adjusted for baseline utility.

Sensitivity analysis assessing the broader societal costs indicated that night splinting was associated with more time off work and higher productivity costs (appendix). The results also show that corticosteroid injection was cost-effective (£2711 per QALY gained) from a health-care perspective and also cost-effective when the crosswalk tariff was used to estimate EQ-5D scores. Cost-effectiveness analysis using the BCTQ score indicates that injection is associated with an ICER of £186 per unit reduction in BCTQ score (appendix).

## Discussion

To our knowledge, this trial is the largest randomised comparison of the short-term and medium-term effectiveness of corticosteroid injection versus night splint for the treatment of carpal tunnel syndrome and the first to be done in a primary care setting; where most patients presenting with mild or moderate symptoms are managed. The results show that at 6 weeks, across all primary and secondary outcome measures analysed, a single injection of 20 mg methylprednisolone acetate led to significantly greater improvements in pain and function than night splints and that these improvements were largely sustained over 6 months (with no difference between groups), which is beyond the active life of methylprednisolone acetate. As a result of further improvements in the night splint group, differences at 6 months were small. The more rapid improvement associated with corticosteroid injection also leads to this treatment being more cost-effective from both an NHS and societal perspective. These 6-month results add to the current inconsistent evidence of only short-term comparative effectiveness of corticosteroid injection versus night splints.^13^, ^14^ Furthermore, we reported only known and expected adverse reactions. Methylprednisolone acetate has been widely used for many years in standard practice in both primary and secondary care and has a very well-established safety profile.

A minimally important clinical difference has not been published for the overall BCTQ for a comparison between these two interventions in primary care. We proposed a minimally important clinical difference based on the results of two available primary care-based studies^34^, ^35^ (at the time of the sample size calculation in 2012) that separately investigated corticosteroid injections and splints and were therefore only indicative for defining the threshold for our study. An 11% difference in overall BCTQ scores was achieved against an anticipated 15% difference and a 0·32 effect size against an anticipated effect size of 0·45 (table 2). However, the pooled SD of 0·786 is smaller than the SD used in the sample size calculation (1·0). Dividing the treatment effect (–0·32) by 0·786 gives an effect size of 0·41, which is approximately equal to the standardised difference of 0·45 used in the sample size calculation. Given this and that findings were consistent across all primary and secondary outcomes with a more rapid improvement of symptoms with injection, we feel the results might be considered sufficiently clinically important by patients with mild–severe symptoms of carpal tunnel syndrome.

No consensus exists for the optimal dose or choice of corticosteroid to inject for carpal tunnel syndrome. One study based in secondary care^36^ found that higher doses of corticosteroid (60 mg methylprednisolone acetate) resulted in more patients being free from clinically significant symptoms than lower doses (20 mg or 40 mg) at 6 months (success of 73% for 60 mg, 56% for 40 mg, and 53% for 20 mg), but there was no evidence of longer-term benefits at 12 months. In a second study, again in secondary care and in patients in whom splinting had already failed, improvements in symptom severity scores at 10 weeks were also greater in patients who received 80 mg and 40 mg of methylprednisolone (difference in change from baseline −0·64, 95% CI −1·06 to −0·21; p=0·003) than in those who received placebo (–0·88, −1·30 to −0·46; p<0·001), but differences were not significant at 1 year.^12^ Our study shows that a single injection of a relatively low dose of 20 mg methylprednisolone acetate (which reflects a consensus regarding usual practice for administering corticosteroid injection for carpal tunnel syndrome in primary care) produced a more rapid improvement of mild or moderate symptoms than use of night splints.

Landmark-guided (also known as blind) corticosteroid injection was used in the trial as this technique is the standard used in primary care where access to ultrasound is usually rare. Unguided corticosteroid injections have been shown to be of similar efficacy to ultrasound-guided injections in reducing symptoms and improving function and electrophysiological findings in carpal tunnel syndrome.^37^

Carpal tunnel syndrome is routinely classified as mild, moderate, or severe, although criteria for this categorisation are not well established and there is lack of an accepted gold standard for diagnosis particularly in relation to the inclusion of electrophysiological tests. In some countries, including the UK, primary care access to nerve conduction studies is variable or limited. As such, these investigations are usually reserved for indeterminate diagnoses and are not always required routinely in primary care for mild or moderate cases to guide decisions regarding initial conservative treatments.^6^ For the purposes of this trial, we felt it was important to develop a tool to standardise the diagnosis of carpal tunnel syndrome presenting in primary care. A full description of the design of this tool has been published elsewhere.^16^

Strengths of our trial include the large sample size, length of follow-up, and full health economic analysis. To answer our pragmatic research question regarding comparative effectiveness, it was crucial to do the trial in a setting very close to routine primary care. To optimise the generalisability of the findings, maximise recruitment, and achieve realistic recruitment targets, we decided to also recruit from community-based musculoskeletal services, which receive direct referrals from multiple GPs who themselves do not inject patients with carpal tunnel syndrome. The characteristics of our trial population were similar to eligible non-participants, which strengthens the generalisability of our findings, with most participants having their first episode of carpal tunnel syndrome with symptoms for at least 3 months. Trial participants and clinicians were not masked to treatment allocation, which is common to many trials of non-pharmacological interventions, and was inherent to the research question and pragmatic design of our trial. Limitations of our trial include the absence of a no-treatment control group, or a group receiving both interventions, which is not uncommon in primary care. We felt it was important to offer treatment to all participants consulting for mild or moderate symptoms, and adding a third group would have increased sample size and compromised the feasibility of the trial in a primary care setting. A clinical assessment was not included at follow-up so we are unable to comment on changes in clinical findings. The recruitment period ended before randomisation of the target of 240 participants, which did not compromise statistical power because of high follow-up, but did mean that the block randomisation sequence was not completed, leading to a minor imbalance in the number of participants in the treatment groups (n=2).

In summary, a single corticosteroid injection shows superior clinical effectiveness at 6 weeks and is cost-effective over 6 months compared with night resting splints, which should make it the treatment of choice for rapid and sustained symptom response in mild or moderate carpal tunnel syndrome presenting in primary care. These findings inform evidence-based treatment choices for GPs and clinicians in community musculoskeletal services.
