# Supplementary material for: The clinical and cost-effectiveness of corticosteroid injection versus night splints for carpal tunnel syndrome (INSTINCTS trial): an open-label, parallel group, randomised controlled trial
Source: Lancet. 2018 Oct 20;392(10156):1423–33. doi: 10.1016/S0140-6736(18)31572-1 (PMC6196880; doi:10.1016/S0140-6736(18)31572-1)
Supplement: Supplementary appendix [file mmc1.pdf]

THE LANCET

Supplementary appendix

This appendix formed part of the original submission and has been peer reviewed. We post it as supplied by the authors.

Supplement to: Chesterton LS, Blagojevic-Bucknall M, Burton C, et al. The clinical and cost-effectiveness of corticosteroid injection versus night splints for carpal tunnel syndrome (INSTINCTS trial): an open label, parallel group, randomised controlled trial. *Lancet* 2018; **392:** 1423–33.

Supplementary file 1: Mean (SD) NHS resource utilisation over 6 months (Complete case analysis)

	Injection N=95	Night splint N=96	Difference (CI)
		Health Professionals	
GP visits	0.421 (0.807)	0.656 (1.204)	-0.24 (-0.54, 0.05)
Nurse visits	0.026 (0.207)	0.051 (0.389)	-0.03 (-0.16, 0.04)
Physiotherapist visits	0.074 (0.550)	0.135 (0.690)	-0.06 (-0.24, 0.11)
Surgeon visit)	0.126 (0.419)	0.135 (0.450)	-0.009 (-0.14, 0.10)
Rheumatologist visit	0.137 (1.048)	0.125 (0.441)	0.01 (-0.15, 0.35)
Acupuncturist visit	0	0.042 (0.408)	-0.04 (-0.14, 0)
Occupational therapist visit	0.021 (0.144)	0.083 (0.496)	-0.06 (-0.19, 0.02)
Other health professionals	0.042 (0.249)	0.135 (0.473)	-0.09 (-0.20, 0.01)
		Investigations and interventions	
X-ray	0.011 (0.103)	0.052 (0.266)	-0.04 (-0.11, 0.01)
Ultrasound	0	0.021 (0.144)	-0.02 (-0.06, 0)
MRI scan	0.021 (0.144)	0.010 (0.102)	0.01 (-0.02, 0.05)
Blood test	0.021 (0.205)	0.104 (0.571)	-0.08 (-0.20, 0.02)

Nerve studies	0.074 (0.300)	0.073 (0.261)	0.001 (-0.06, 0.07)
Wrist splint	0.063 (0.245)	0.250 (0.481)	-0.19 (-0.24, -0.06)
Wrist injection	0.295 (0.503)	0.208 (0.501)	0.09 (-0.05, 0.18)
Wrist exercise	0.021 (0.144)	0.020 (0.144)	0.001 (-0.35, 0.03)

Supplementary file 2: Mean (SD) NHS costs over 6 months (£) (Complete case analysis)

	Injection N=95	Night splint N=96	Difference (CI)
		Health Professionals	
GP visits	12.21 (23.39)	19.03 (34.91)	-6.82 (-15.10, 1.15)
Nurse visits	0.57 (4.11)	1.13 (7.75)	-0.56 (-2.81, 0.93)
Physiotherapist visits	3.32 (24.77)	6.09 (31.05)	-2.78 (-11.61, 4.72)
Surgeon visit	8.21 (27.22)	8.80 (29.25)	-0.59 (-9.23, 7.40)
Rheumatologist visit	6.15 (47.16)	5.63 (19.86)	0.52 (-6.89, 13.17)
Acupuncturist visit	0	1.88 (18.37)	-1.88 (-6.99, 0)
Occupational therapist visit	0.88 (6.06)	3.50 (20.81)	-2.62 (-7.67, 0.92)
Other health professionals	1.63 (9.24)	2.14 (12.31)	-0.51 (-3.68, 2.42)
		Investigations and interven tions	
Blood test	0.13 (1.23)	0.63 (3.43)	-0.50 (-1.49, 0.05)
Ultrasound cost	0	1.14 (7.90)	-1.14 (-3.16, 0)
MRI scan cost	3.45 (23.67)	1.71 (16.74)	1.74 (-3.52, 8.28)
Surgery cost	132.12 (470.92)	112.06 (436.29)	20.05 (-101.69, 160.05)
Additional Wrist splint cost	3.15 (12.23)	12.5 (24.06)	-9.35 (-5.55, -1.68)
Additional Wrist Injection cost	15.62 (26.63)	11.04 (26.55)	4.58 (-2.81, 11.36)
Wrist exercise cost	13.26 (22.62)	9.38 (22.54)	3.89 (-2.51, 10.14)
Nerve conduction cost	12.16 (49.57)	12.03 (43.16)	0.13 (-12.86, 14.47)
Prescribed medication cost	1.30 (5.61)	1.92 (7.68)	-0.61 (-2.83, 0.99)
Intervention cost[a]	138.94 (69.17)	94.02 (65.66)	44.92 (24.83, 62.13)
		Total Costs (complete cases)	

Total cost (NHS)	353.48 (512.85)	306.42 (524.51)	47.06 (-104.84, 187.31)
		Total Costs (imputed analysis)	
	N=116	N=118	
Total cost (NHS)	346.78 (467.97)	313.24 (480.84)	33.54 (-94.57, 145.59)

[a] Costs associated with the intervention took into account whether the patient was seen by a GP or physio and whether they were seen in the GP practice or interface clinic. Unit costs associated with the intervention are as follows: Injection by GP in interface clinic (£191); Injection by GP in GP practice (£207); Injection by Physio in interface clinic (£53); Injection by Physio in GP practice (£69); Splinting by GP in interface clinic (£188); Splinting by GP in GP practice (£204); Splinting by Physio in interface clinic (£50); Splinting by Physio in GP practice (£66)

Supplementary file 3: Mean (SD) Health outcomes over 6 months

	Injection<br>N=116	Night splint<br>N=118	Difference (CI) (Injection – Night splint)
		EQ-5D	
EQ-5D baseline	0.764 (0.190)	0.784 (0.190)	-0.02 (-0.07, 0.03))
EQ-5D 6 weeks	0.794 (0.200)	0.781 (0.205)	0.01 (-0.04, 0.06)
EQ-5D 6 months	0.819 (0.161)	0.826 (0.174)	-0.007 (-0.05, 0.04)
		Total QALYs	
QALYs (Unadjusted)	0.400 (0.083)	0.399 (0.086)	0.001 (-0.02, 0.02)
QALYs (Adjusted)[a]	0.404	0.396	0.008 (-0.01, 0.02)

[a] Adjusted for baseline utility

Supplementary file 4: Sensitivity analysis: time off work over 6 months. Mean (SD) per patient (Complete cases)

	Injection N=95	Night splinting N=96	Difference (CI) (Injection - Night splint)
		Broader societal costs	
Performance at work 6 weeks [a]	0.18 (0.95)	0.26 (1.25)	-0.14 (-0.47, 0.18)
Performance at work 6 months [a]	0.87 (1.86)	1.32 (2.44)	-0.36 (-0.88, 0.16)
Days off-work 6 weeks	0.07 (0.51)	0.05 (0.30)	0.02 (-0.10, 0.14)
Days off-work 6 months	0.58 (3.88)	1.42 (7.85)	-0.84 (-2.64, 0.94)
Productivity costs [b]	50.42 (310.99)	93.76 (574.85)	-43.34 (-202.72, 71.34)
			-33.70 (-167.36, 99.96) [c]

[a] Mean performance at work on a scale of 0 to 10 where 0 indicates work performance not affected [b] Productivity costs obtained from days off-work at 6 weeks and 6 months
[c] Adjusted for employment at baseline

Supplementary file 5: Sensitivity analysis

Mean cost difference over 6 months (Injection – Night splint)	Mean QALY difference over 6 months (Injection – Night splint)	ICER
Healthcare perspective		
£21.69	0.008	£2,711 per QALY gained
EQ-5D using cross-walk tariff (NHS perspective)		
£33.54	0.004	£8,385 per QALY gained
Cost-effectiveness using the Boston CTS (NHS perspective)		
Mean cost difference over 6 months (Injection – Night splint)	Mean Boston CTS score difference over 6 months (Injection – Night splint)	ICER
£33.54	-0.18[a]	£186 per unit reduction in Boston CTS score

[a] overall Boston CTS score over 6 months controlling for baseline score, age and sex

Baseline characteristics of participants followed-up and lost to follow-up at 6 weeks

Characteristics	Completed 6 week follow-up N=217	Lost to follow-up at 6 weeks N=17
Treatment Indicator		
A	109 (50.2)	9 (52.9)
B	108 (49.8)	8 (47.1)
Demographics and health		
Age (Mean (SD))	53.3 (15.8)	40.6 (13.4)
Gender		
Male	72 (33.2)	8 (47.1)
Female	145 (66.8)	9 (52.9)
In a currently paid job		
Yes	121 (55.8)	11 (64.7)
No	92 (42.4)	5 (29.4)
Missing	4 (1.8)	1 (5.9)
Alcohol consumption		
Daily or most days	21 (9.7)	1 (5.9)
Once or twice a week	77 (35.5)	6 (35.3)
Once or twice a month	44 (20.3)	3 (17.6)
Once or twice a year	32 (14.7)	2 (11.8)
Never	40 (18.4)	4 (23.5)
Missing	3 (1.4)	1 (5.9)
Smoking status		
Never smoked	101 (46.5)	9 (52.9)
Previously smoked	82 (37.8)	5 (29.4)
Current smoker	31 (14.3)	1 (5.9)
Missing	3 (1.4)	2 (11.8)
Pain anywhere else		
Yes	137 (63.1)	9 (52.9)
No	77 (35.5)	7 (41.2)
Missing	3 (1.4)	1 (5.9)
Current CTS		
First time diagnosed with CTS		
Yes	185 (85.3)	14 (82.4)
No	29 (13.4)	2 (11.8)
Missing	3 (1.4)	1 (5.9)
How did your hand or wrist problems start		
Suddenly	46 (21.2)	4 (23.5)
Gradually	166 (76.5)	12 (70.6)
Missing	5 (2.3)	1 (5.9)
Currently taking pain relief		
Yes	64 (29.5)	6 (35.3)
No	150 (69.1)	10 (58.8)
Missing	3 (1.4)	1 (5.88)
How long had your current hand or wrist problems for		
<3 months	32 (14.7)	4 (23.5)
3-6 months	68 (31.3)	2 (11.8)
6 months – 1 year	44 (20.3)	5 (29.4)
>1 year	68 (31.3)	5 (29.4)
Missing	5 (2.3)	1 (5.9)
Which hand or wrist problems do you have problems with		
Right	65 (30.0)	8 (47.1)
Left	37 (17.1)	2 (11.8)
Both	110 (50.7)	6 (35.3)
Missing	5 (2.3)	1 (5.9)

Hand or wrist problems has major consequences on my life		
Strongly disagree	14 (6.5)	0 (0.0)
Disagree	39 (18.0)	2 (11.8)
Neither agree nor disagree	51 (23.5)	5 (29.4)
Agree	71 (32.7)	7 (41.2)
Strongly agree	38 (17.5)	2 (11.8)
Missing	4 (1.8)	1 (5.9)
My hand or wrist problem affects me emotionally		
Strongly disagree	26 (12.0)	2 (11.8)
Disagree	33 (15.2)	3 (17.6)
Neither agree nor disagree	36 (16.6)	2 (11.8)
Agree	79 (36.4)	6 (35.3)
Strongly agree	39 (18.0)	3 (17.6)
Missing	4 (1.8)	1 (5.9)
Outcomes		
Overall Boston CTS (Mean (SD))	2.6 (0.7)	2.9 (0.3)
Symptom severity (Mean (SD))	2.9 (0.6)	3.2 (0.3)
Functional status (Mean (SD))	2.2 (0.9)	2.5 (0.4)
Hand wrist symptom severity	6.2 (2.1)	7.1 (1.4)
Insomnia		
Yes	116 (53.5)	10 (58.8)
No	97 (44.7)	6 (35.3)
Missing	4 (1.8)	1 (5.9)

Baseline characteristics of participants followed-up and lost to follow-up at 6 months

Characteristics	Completed 6 month follow-up N=193	Lost to follow-up at 6 months N=41
Treatment Indicator		
A	97 (50.3)	21 (51.2)
B	96 (49.7)	20 (48.8)
Demographics and health		
Age (Mean (SD))	54.3 (15.7)	43.4 (14.0)
Gender		
Male	65 (33.7)	15 (36.6)
Female	128 (66.3)	26 (63.4)
In a currently paid job		
Yes	105 (54.4)	27 (65.9)
No	84 (43.5)	13 (31.7)
Missing	4 (2.1)	1 (2.4)
Alcohol consumption		
Daily or most days	18 (9.3)	4 (9.8)
Once or twice a week	67 (34.7)	16 (39.0)
Once or twice a month	40 (20.7)	7 (17.1)
Once or twice a year	29 (15.0)	5 (12.2)
Never	36 (18.7)	8 (19.5)
Missing		
Smoking status		
Never smoked	89 (46.1)	21 (51.2)
Previously smoked	74 (38.3)	13 (31.7)
Current smoker	26 (13.5)	6 (14.6)
Missing	4 (2.1)	1 (2.4)
Pain anywhere else		
Yes	119 (61.7)	27 (65.9)
No	71 (36.8)	13 (31.7)
Missing	3 (1.6)	1 (2.4)
Current CTS		
First time diagnosed with CTS		
Yes	167 (86.5)	32 (78.0)
No	23 (11.9)	8 (19.5)
Missing	3 (1.6)	1 (2.4)
How did your hand or wrist problems start		
Suddenly	38 (19.7)	12 (29.3)
Gradually	150 (77.7)	28 (68.3)
Missing	5 (2.6)	1 (2.4)
Currently taking pain relief		
Yes	52 (26.9)	18 (43.9)
No	138 (71.5)	22 (53.7)
Missing	3 (1.6)	1 (2.4)
How long had your current hand or wrist problems for		
<3 months	29 (15.0)	7 (17.1)
3-6 months	63 (32.6)	7 (17.1)
6 months – 1 year	39 (20.2)	10 (24.4)
>1 year	57 (29.5)	16 (39.0)
Missing	5 (2.6)	1 (2.4)
Which hand or wrist problems do you have problems with		
Right	59 (30.6)	14 (34.1)
Left	33 (17.1)	6 (14.6)
Both	96 (49.7)	20 (48.8)
Missing	5 (2.6)	1 (2.4)

Hand or wrist problems has major consequences on my life		
Strongly disagree	14 (7.3)	0 (0.0)
Disagree	37 (19.2)	4 (9.8)
Neither agree nor disagree	46 (23.8)	10 (24.4)
Agree	65 (33.7)	13 (31.7)
Strongly agree	27 (14.0)	13 (31.7)
Missing	4 (2.1)	1 (2.4)
My hand or wrist problem affects me emotionally		
Strongly disagree	26 (13.5)	2 (4.9)
Disagree	30 (15.5)	6 (14.6)
Neither agree nor disagree	35 (18.1)	3 (7.3)
Agree	67 (34.7)	18 (43.9)
Strongly agree	31 (16.1)	11 (26.8)
Missing	4 (2.1)	1 (2.4)
Outcomes		
Overall Boston CTS (Mean (SD))	2.6 (0.7)	3.0 (0.6)
Symptom severity (Mean (SD))	2.9 (0.6)	3.3 (0.5)
Functional status (Mean (SD))	2.2 (0.9)	2.7 (0.8)
Hand wrist symptom severity	6.0 (2.2)	7.1 (1.7)
Insomnia		
Yes	88 (45.6)	15 (36.6)
No	101 (52.3)	25 (61.0)
Missing	4 (2.1)	1 (2.4)

Boston Carpal Tunnel Syndrome Questionnaire (BCTQ)

(一) Symptom severity scale (11 items)

	1	2	3	4	5
1. How severe is the hand or wrist pain that you have at night?	Normal	Slight	Medium	Severe	Very serious
2. How often did hand or wrist pain wake you up during a typical night in the past two weeks?	Normal	Once	2 to 3 times	4 to 5 times	More than 5 times
3. Do you typically have pain in your hand or wrist during the daytime?	No pain	Slight	Medium	Severe	Very serious
4. How often do you have hand or wrist pain during daytime?	Normal	1-2 times / day	3-5 times / day	More than 5 times	Continued
5. How long on average does an episode of pain last during the daytime?	Normal	＜10minutes	10~60 Continued	＞60minutes	Continued
6. Do you have numbness (loss of sensation) in your hand?	Normal	Slight	Medium	Severe	Very serious
7. Do you have weakness in your hand or wrist?	Normal	Slight	Medium	Severe	Very serious
8. Do you have tingling sensations in your hand?	Normal	Slight	Medium	Severe	Very serious
9. How severe is numbness (loss of sensation) or tingling at night?	Normal	Slight	Medium	Severe	Very serious
10. How often did hand numbness or tingling wake you up during a typical night during the past two weeks?	Normal	Once	2 to 3 times	4 to 5 times	More than 5 times
11. Do you have difficulty with the grasping and use of small objects such as keys or pens?	Without difficulty	Little difficulty	Moderately difficulty	Very difficulty	Very difficult

(二) Functional status scale (8 items)：

	No difficulty	Little difficulty	Moderate difficulty	Intense difficulty	Cannot perform the activity at all due to hands and wrists symptoms
Writing	1	2	3	4	5
Buttoning of clothes	1	2	3	4	5
Holding a book while reading	1	2	3	4	5
Gripping of a telephone handle	1	2	3	4	5
Opening of jars	1	2	3	4	5
Household chores	1	2	3	4	5
Carrying of grocery basket	1	2	3	4	5
Bathing and dressing	1	2	3	4	5

Study ID:

INSTinCTS

INjection versus SplinTing in Carpal Tunnel Syndrome

Pre-Treatment Questionnaire

Version 4.0, 7th November 2014

The answers you give in this questionnaire will be treated in the strictest of confidence

Thank you for your help with this study

EudraCT No:2013-001435-48
REC No:13/NW/0280

Instructionsforthis

questionnaire

The aim of this questionnaire is to find out about your hand or wrist problem and the impact it has on your life.

Full details about this study are available in the information sheet provided separately.

Most of the questions can be answered either by putting a tick in a box next to your answer or by circling a number, for example:

☒ Yes ☐ No

OR

(1) 2 3

Please write in BLOCK CAPITALS where appropriate.

- **Please answer all of the questions**, even if you are not completely sure of your answer. There are no 'correct' or 'incorrect' answers
- Some questions do look similar but it is important that you answer them all as as accurately as possible as this will help our research
- When you have finished, please check that you have answered all of the questions and give the questionnaire back to the doctor, research nurse or physiotherapist before you leave

Section A *This section asks general questions about you*

1. Are you: Male ☐ Female ☐

2. What is your date of birth? ☐☐ / ☐☐ / ☐☐☐☐ *(for example, if you were born on 5th June 1964, this would be entered as 05/06/1964)*

3. Today's date: ☐☐ / ☐☐ / ☐☐☐☐

Section B *This section is about whether you have had carpal tunnel syndrome before*

1. Is this the first time that you have been diagnosed with carpal tunnel syndrome?

Yes ☐ *Please go to Section C on page 6*

No ☐ ***Please continue with question 2***

2. How many times have you had carpal tunnel syndrome in the past (not including this time)?

1 ☐ 2 ☐ 3 ☐ More than 3 ☐

3. Which hand or wrist has been affected by carpal tunnel syndrome in the past?

Right ☐ Left ☐ Both ☐

Section B Continued...

4. Which of the following treatments have you received for your carpal tunnel syndrome in the past? (***Please tick all that apply***)

   a. None ☐ *Please go to question 6*

   b. Steroid injection ☐

   c. Wrist splints ☐

   d. Carpal tunnel decompression (surgery) ☐

   e. Ultrasound ☐

   f. Exercises ☐

   g. Vitamin supplements ☐

   h. Changes in the work place ☐

   i. Other please state in the box [ ]

5. For each treatment you have received in the past detailed in Question 4, please state for each one how useful it has been

	Treatment	Of great help	Of some help	Of little help	Of no help
a.		☐	☐	☐	☐
b.		☐	☐	☐	☐
c.		☐	☐	☐	☐
d.		☐	☐	☐	☐

6. Have you ever had a steroid injection into a joint other than your wrist?

   Yes ☐ ***Please go to question 7***

   No ☐ ***Please go to Section C on page 6***

7. How useful did you find the injection into your other joint?

Of great help	Of some help	Of little help	Of no help
☐	☐	☐	☐

Section C

This section is about your current episode of hand or wrist problems. If you have problems with both hands or wrists, please answer these questions about the hand or wrist that is going to be treated in the trial.

1a. Which hand or wrist do you have problems with?

Right ☐ Left ☐ Both ☐

1b. **If** both hands, which hand is worse?

Right ☐ Left ☐ No difference ☐

2. Which is your dominant hand (e.g. the hand that you write with)?

Right ☐ Left ☐

3. How long have you had your current hand or wrist problems for? ***(you do not need to be exact, please tick one box closest to your answer)***

Less than 3 months ☐

Between 3 months and 6 months ☐

Between 6 months and 1 year ☐

More than 1 year ☐

4. How did your hand or wrist problems start? (Please tick one box)

Suddenly: symptoms developed quickly within a few days ☐

Gradually: symptoms developed more slowly over weeks to months ☐

5. Is there a particular hand or wrist position which causes your hand or wrist problem? (Please tick one box)

Yes ☐ No ☐

6. In the **last 24 hours**, on **average** how intense were your hand or wrist symptoms, on a 0 to 10 scale, where 0 is 'no symptoms' and 10 is 'worst symptoms imaginable'? ***(Please circle one number)***

**No symptoms**										**Worst symptoms imaginable**
0	1	2	3	4	5	6	7	8	9	10

Section C continued...

7. Are you currently **using any pain relief** for your hand or wrist problems?

Yes ☐ ***Please go to question 8***

No ☐ ***Please go to question 9***

8. Approximately, **how often** are you taking pain relief? (***Please write a number in each box***)

Number of times per day ☐

Number of days per week ☐

Number of weeks in a month ☐

The following questions refer to your symptoms **for a typical twenty-four hour period** during the past two weeks (***circle one answer to each question***).

9. How severe is the hand or wrist pain that you have at night?

1 I do not have any pain
2 Mild pain
3 Moderate pain
4 Severe pain
5 Very severe pain

10. How often did hand or wrist pain wake you up during a typical night in the past two weeks?

1 Never
2 Once
3 Two or three times
4 Four or five times
5 More than five times

11. Do you typically have pain in your hand or wrist during the daytime?

1 I have never had pain during the day
2 I have mild pain during the day
3 I have moderate pain during the day
4 I have severe pain during the day
5 I have very severe pain during the day

Section C continued...

The following questions refer to your symptoms **for a typical twenty-four hour period** during the past two weeks ***(circle one answer to each question).***

12. How often do you have hand or wrist pain during the daytime?

1 Never
2 Once or twice a day
3 Three to five times a day
4 More than five times a day
5 The pain is constant

13. How long, on average, does an episode of pain last during the daytime?

1 I never get pain during the day
2 Less than 10 minutes
3 10 to 60 minutes
4 Greater than 60 minutes
5 The pain is constant throughout the day

14. Do you have numbness (loss of sensation) in your hand?

1 No
2 I have mild numbness
3 I have moderate numbness
4 I have severe numbness
5 I have very severe numbness

15. Do you have weakness in your hand or wrist?

1 No weakness
2 Mild weakness
3 Moderate weakness
4 Severe weakness
5 Very severe weakness

Section C continued...

The following questions refer to your symptoms **for a typical twenty-four hour period** during the past two weeks ***(circle one answer to each question)*.**

16. Do you have tingling sensations in your hand?

1 No tingling

2 Mild tingling

3 Moderate tingling

4 Severe tingling

5 Very severe tingling

17. How severe is numbness (loss of sensation) or tingling at night?

1 I have no numbness or tingling at night

2 Mild

3 Moderate

4 Severe

5 Very severe

18. How often did hand numbness or tingling wake you up during a typical night during the past two weeks?

1 Never

2 Once

3 Two or three times

4 Four or five times

5 More than five times

19. Do you have difficulty with the grasping and use of small objects such as keys or pens?

1 No difficulty

2 Mild difficulty

3 Moderate difficulty

4 Severe difficulty

5 Very severe difficulty

Section C continued...

20. On a typical day during the past two weeks have your hand or wrist symptoms caused you to have any difficulty doing the activities listed below? ***Please circle one number on each line that best describes your ability to do the activity.***

**Activity**	**No difficulty**	**Mild difficulty**	**Moderate difficulty**	**Severe difficulty**	**Cannot do at all due to hand or wrist symptoms**
a. Writing	1	2	3	4	5
b. Buttoning of clothes	1	2	3	4	5
c. Holding a book while reading	1	2	3	4	5
d. Gripping of a telephone handle	1	2	3	4	5
e. Opening of jars	1	2	3	4	5
f. Household chores	1	2	3	4	5
g. Carrying of grocery bags	1	2	3	4	5
h. Bathing and dressing	1	2	3	4	5

Section D This section asks how your hand or wrist problems may be affecting your sleep

Thinking over the **past 6 weeks** did you: ***(please put a tick in one box on each line)***

1. Have trouble falling asleep because of your hand and wrist problems?

☐ Not at all ☐ On some nights ☐ On most nights

2. Wake up several times per night because of your hand and wrist problems?

☐ Not at all ☐ On some nights ☐ On most nights

3. Have trouble staying asleep because of your hand and wrist problems?

☐ Not at all ☐ On some nights ☐ On most nights

4. Wake up after your usual amount of sleep feeling tired and worn out?

☐ Not at all ☐ On some nights ☐ On most nights

Section E *This section asks how your hand or wrist problems affect your work*

1. Are you currently in a paid job?

Yes ☐ *Please go to question 3a) on page 12*

No ☐ *Please go to question 2*

2. Which of the following best describes your current situation? (Please tick ***all that apply***)

Retired ☐

Student ☐

Looking after children / home ☐

Unemployed ☐

Voluntary worker ☐

Please now go to section F on page 14

3 a) What is your current paid job title?

b) What do the firm / organisation you work for mainly make or do?

c) What do you mainly do in your job?

4. How would you best describe your typical working week in terms of the hours spent at work? (Please put ***a tick in one box***)

Working full time (35 hours or more per week) ☐

Working part time (working less than 35 hours per week) ☐

5. Which of the following best describes your current situation in respect of your job? (Pleas***e put a tick in one box***)

Doing my usual job ☐
On paid annual leave / holiday ☐
Working fewer hours ☐
Doing lighter duties ☐
On paid sick leave ☐
On unpaid leave ☐

6. If you are not doing your usual job, is this because of your hand or wrist problems?

Yes ☐ No ☐

7. On average to what extent have your hand or wrist problems affected your performance at work over the past month?

Please circle one number on the 0 to 10 scale, where 0 is 'Not at all' and 10 is 'The problems are so bad I am unable to do my job'

**Not at all**										**The problems are so bad I am unable to do my job**
0	1	2	3	4	5	6	7	8	9	10

Section E continued...

8. Have you taken time off work during the past 6 months for your hand or wrist problem?

   Yes ☐ *Please go to question 9*

   No ☐ *Please go to Section F*

9. Please ***write in the box*** the number of days, you were absent from work due to your hand or wrist problem in the past 6 months ☐

10. Please indicate how much you agree or disagree with the following statement by putting a tick in one box

	Strongly disagree	Disagree	Agree	Strongly agree
I feel that my welfare is important to my employer	☐	☐	☐	☐

Section F This section is about what you expect from your treatment, the sort of treatment you might prefer if you had a choice and your own personal views on how you see your hand or wrist problems

1. As explained in the information sheet, you will have an equal chance of being given one of two treatments either a wrist injection or a night splint, but you will not be able to choose. However, if you were to have a choice, would you prefer one treatment over the other? (Pleas***e put a tick in one box only***)

   I would strongly prefer a wrist injection ☐

   I would somewhat prefer a wrist injection ☐

   I have no preference ☐

   I would somewhat prefer night splints ☐

   I would strongly prefer night splints ☐

2. If you were to receive a **wrist injection,** would you expect your symptoms to improve within 6 weeks following treatment? (Please ***tick one box)***

   ☐ Yes ☐ No ☐ Not sure

3. If you were to receive a **night splint**, would you expect your symptoms to improve within 6 weeks following treatment? (Please ***tick one box****)*

   ☐ Yes ☐ No ☐ Not sure

Section F continued...

4. Please indicate how much you agree or disagree with the following statements by putting a tick in one box on each line

	Strongly disagree	Disagree	Neither agree nor disagree	Agree	Strongly agree
a. My hand or wrist problem will last for a long time	☐	☐	☐	☐	☐
b. My hand or wrist problem has major consequences on my life	☐	☐	☐	☐	☐
c. There is a lot that I can do to control my hand or wrist problems	☐	☐	☐	☐	☐
d. What I do can determine whether my hand or wrist problem gets better or worse	☐	☐	☐	☐	☐
e. Treatment can control my hand or wrist problem	☐	☐	☐	☐	☐
f. My hand or wrist problem affects me emotionally (e.g. it makes me feel frustrated, anxious, angry, afraid, upset or depresssed)	☐	☐	☐	☐	☐

Section G This section asks about your quality of life

Under each heading, ***please tick the ONE box*** that best describes your health TODAY.

1. **Mobility**
   - ☐ I have no problems in walking about
   - ☐ I have slight problems in walking about
   - ☐ I have moderate problems in walking about
   - ☐ I have severe problems in walking about
   - ☐ I am unable to walk about
2. **Self-care**
   - ☐ I have no problems washing or dressing myself
   - ☐ I have slight problems washing or dressing myself
   - ☐ I have moderate problems washing or dressing myself
   - ☐ I have severe problems washing or dressing myself
   - ☐ I am unable to wash or dress myself
3. **Usual activities** (e.g. work, study, housework, family or leisure activities)
   - ☐ I have no problems doing my usual activities
   - ☐ I have slight problems doing my usual activities
   - ☐ I have moderate problems doing my usual activities
   - ☐ I have severe problems doing my usual activities
   - ☐ I am unable to do my usual activities
4. **Pain / discomfort**
   - ☐ I have no pain or discomfort
   - ☐ I have slight pain or discomfort
   - ☐ I have moderate pain or discomfort
   - ☐ I have severe pain or discomfort
   - ☐ I have extreme pain or discomfort
5. **Anxiety / depression**
   - ☐ I am not anxious or depressed
   - ☐ I am slightly anxious or depressed
   - ☐ I am moderately anxious or depressed
   - ☐ I am severely anxious or depressed
   - ☐ I am extremely anxious or depressed

Section G continued...

6. During the last month have you often been bothered by feeling down, depressed or hopeless? Yes ☐ No ☐

7. During the last month have you often been bothered by little interest or pleasure in doing things? Yes ☐ No ☐

Section H This section asks about your general health

1. Have you been diagnosed with hypothyroidism (a low / underactive thyroid gland)? Yes ☐ No ☐

2. Have you been diagnosed with diabetes? Yes ☐ No ☐

3 . Apart from your current hand or wrist problem, do you have any other conditions affecting your neck, shoulders or elbows? Yes ☐ No ☐

4. Have you had pain anywhere else (apart from your hand or wrist) in the last month? Yes ☐ No ☐

5. When was the last time you were free of pain (anywhere) for a month or more?

Less than 3 months ago ☐
Between 3 months and 6 months ☐
Between 6 months and 1 year ☐
Between 1 year and 3 years ☐
More than 3 years ☐

6. On average how often do you drink alcohol?

Daily or most days ☐
Once or twice a week ☐
Once or twice a month ☐
Once or twice a year ☐
Never ☐

7. What is your current smoking status?

Never smoked ☐ Previously smoked (ex-smoker) ☐ Current smoker ☐

End of Questionnaire

Thank you for taking the time to fill in this questionnaire, your answers will be very useful to us.

Please check that you have answered all of the questions and return the questionnaire to your doctor, physiotherapist or nurse.
